# Minimally Invasive Supraorbital vs. Traditional Pterional Approaches in Unruptured Aneurysm Surgery: Evaluating Risks and Results

**DOI:** 10.3390/brainsci15121315

**Published:** 2025-12-09

**Authors:** Anna Brunner, Marlene Rainer, Uschi Pongratz, Klaus Leber, Máté Fehér, Alexander Micko, Stefan Wolfsberger

**Affiliations:** Department of Neurosurgery, Medical University of Graz, 8010 Graz, Austria; anna.brunner@medunigraz.at (A.B.); uschi.pongratz@stud.medunigraz.at (U.P.);

**Keywords:** pterional craniotomy, supraorbital craniotomy, unruptured intracranial aneurysms

## Abstract

**Background/Objectives:** Intracranial aneurysms affect 3–5% of the population and are associated with high morbidity and mortality, particularly after rupture. Treatment options for unruptured aneurysms include microsurgical clipping, with the pterional and supraorbital approaches commonly employed. This study compares these two approaches regarding complications and outcomes. **Methods:** A retrospective analysis was conducted on 241 patients treated between 2004 and 2023 at the University Hospital of Graz. Patients underwent microsurgical clipping via the pterional (*n* = 170) or supraorbital (*n* = 71) approach, chosen according to aneurysm characteristics and surgeon preference. Data on demographics, aneurysm location and size, intraoperative complications, postoperative outcomes, and follow-up were evaluated. **Results:** The pterional approach was predominantly used for middle cerebral artery (MCA) aneurysms (79.2%), while the supraorbital approach was more frequently applied for internal carotid artery (ICA) and anterior communicating artery (ACOM) aneurysms. Aneurysms treated via the pterional approach were significantly larger (mean width 6.88 mm vs. 5.04 mm; *p* < 0.01). Severe intraoperative complications, including aneurysm rupture, were significantly more common in the supraorbital group (26.8% vs. 8.8%; *p* < 0.001). Postoperative hypo-/anosmia occurred more often after the supraorbital approach (8.5% vs. 1.8%; *p* = 0.013), while temporalis muscle atrophy (11.9% vs. 1.8%; *p* = 0.029) and chewing difficulties (19.5% vs. 1.8%; *p* = 0.002) were more frequent after the pterional approach. The supraorbital group had a shorter hospital stay (7.96 vs. 8.76 days; *p* = 0.001). No significant differences were found in 30-day mortality (*p* = 0.521). At one-year, functional outcomes assessed by the modified Rankin Scale showed no significant difference (*p* = 0.899). Complete aneurysm occlusion and recurrence rates were also comparable between groups. **Conclusions:** Both approaches provide effective treatment for unruptured aneurysms with favorable long-term outcomes. The pterional approach is associated with increased muscle-related complications, whereas the supraorbital approach carries higher risks of intraoperative complications and olfactory dysfunction. Tailoring the surgical approach based on patient and aneurysm characteristics remains essential.

## 1. Introduction

Intracranial aneurysms are a prevalent condition, affecting about 3–5% of the general population [1,2]. The need for treatment becomes apparent considering that the mortality rate associated with aneurysmal subarachnoid hemorrhage is still notably high, with an 18.4% short-term mortality and a 29% mortality rate over five years [3]. An unfavorable functional outcome (modified Rankin Scale score of 3 to 6) is seen in 42% of these patients one year after suffering from aneurysmal hemorrhage [3].

The main treatment options for intracranial aneurysms include microsurgical clipping and endovascular treatment [4,5]. Although endovascular interventions are less invasive and linked to reduced perioperative morbidity, they come with disadvantages such as higher aneurysm recurrence rates and the requirement for further treatment [6]. Given the growing inclination towards endovascular methods in recent years, there have been attempts to render microsurgical intervention less invasive [7]. This includes the advancement of keyhole approaches, which aim to reduce surgical trauma while preserving the benefit of a more durable occlusion of the aneurysm compared to endovascular treatment [8,9].

In 1975, Yasargil and Fox introduced the pterional approach, which subsequently became the standard method for addressing pathologies of the anterior and middle cranial fossa, and thus, for clipping aneurysms in the anterior circulation [10].

A significant drawback of the pterional craniotomy is the necessary transection of the temporalis muscle, which can result in chewing discomfort during the initial postoperative phase and may cause muscle atrophy over time, leading to aesthetic concerns. Furthermore, there is a risk of damaging the frontal branch of the facial nerve, potentially resulting in partial peripheral facial palsy, which may be enduring and cosmetically troubling [11,12,13].

Over the years, several modifications have been implemented to the approach in order to reduce invasiveness and lessen associated complications [10].

Within this framework, initiatives to create less invasive techniques led to the emergence of keyhole approaches for the clipping of intracranial aneurysms as early as the late 1970s. Expanding on this idea, Perneczky et al. introduced the supraorbital approach in 1998 as a safe and effective alternative for treating intracranial aneurysms, emphasizing its benefits, which include reduced exposure of the brain, decreased brain retraction, and enhanced cosmetic results due to improved preservation of vascular and neural structures in the frontotemporal area [14].

In the subsequent years, both techniques were evaluated in numerous studies, including systematic reviews and meta-analyses, to ascertain their optimal indications for treating both ruptured and unruptured aneurysms. These studies exclusively analyzed mixed cohorts of ruptured and unruptured aneurysms, assessing anatomical exposure, safety, complication rates, and outcomes, yielding somewhat conflicting results [8,14,15,16,17,18,19,20,21].

This study compares the pterional and supraorbital approaches by exclusively focusing on complications in unruptured aneurysms. To our knowledge, this represents the largest study to date that specifically examines non-ruptured aneurysms within this context.

## 2. Materials and Methods

### 2.1. Patients and Methods

Between January 2004 and December 2023, a total of 241 patients with unruptured intracranial aneurysms were treated at the Department of Neurosurgery, University Hospital of Graz. Aneurysms were surgically treated using either a pterional or supraorbital approach.

**Figure 1:**
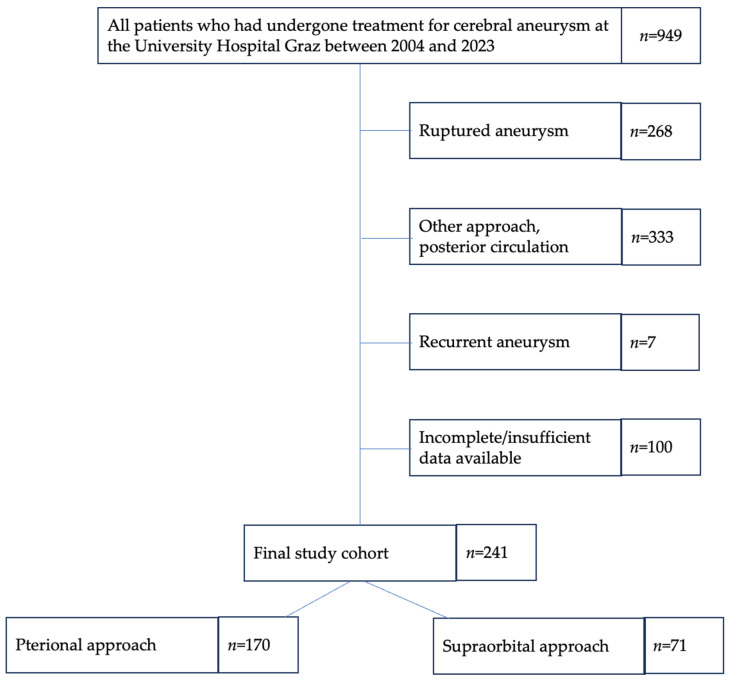
Study flow chart. Abbreviations: *n*, number of patients.

The study flow chart is shown in Figure 1.

### 2.2. Patients

Patients diagnosed with aneurysms using magnetic resonance angiography (MRA) or computed tomography angiography (CTA) were referred to our neurosurgical outpatient clinic. Following evaluation, all patients were informed about their treatment options, which included observation, microsurgical intervention, or endovascular therapy. Treatment decisions, including the chosen modality, were made by a multidisciplinary neurovascular team composed of neurosurgeons, interventional neuroradiologists, and neurologists. The choice of surgical approach—either pterional or supraorbital—was decided by the operating surgeon, taking into account the aneurysm’s location, projection, size, anatomical features, and the patient’s preferences.

### 2.3. Operative Technique

In the following section, the two surgical approaches are described as they are routinely performed at our department.

Pterional approach: The patient is placed in a supine position with the head stabilized in a head clamp. Based on the aneurysm’s location, the head is rotated to a variable degree to the contralateral side. A curvilinear incision is created along the hairline. In certain instances, an interfascial dissection is carried out; alternatively, the temporalis muscle is incised in line with the skin incision, depending on the surgeon’s choice. A craniotomy, roughly 3 × 4 cm in dimension, is conducted following the placement of around three burr holes. The craniotomy is extended in alignment with the frontobasal area, and the sphenoid ridge is partially removed. The dura mater is incised in a semi-circular fashion and retracted downward. One or two retractors are employed; when two are utilized, one is positioned for frontal lobe retraction and the other for temporal lobe retraction.

Supraorbital approach: The patient is placed in a supine position, with the head elevated and slightly tilted downward in the head clamp. Depending on the aneurysm’s position, the head is rotated to the opposite side at varying angles. A C-shaped incision is made in the eyebrow, 2 mm lateral to the supraorbital foramen, extending to the frontozygomatic junction. The muscle (orbicularis oculi) is also cut in a C-shape slightly above the skin incision and retracted with fishhooks. A craniotomy, approximately 2 × 3 cm in size, is then executed using a piezoelectric device. The orbital roof is meticulously dissected and smoothed. The dura mater is incised in a curved manner and retracted downward. A retractor is subsequently positioned to retract the frontal lobe.

Olfactory nerve preservation was not performed as a routine step, and no standardized dissection or mobilization of the nerve was applied.

### 2.4. Outcome

Data regarding the primary and secondary endpoints were gathered retrospectively from patient records. Information on demographics, such as age, sex, mRS at admission, and ASA classification, was obtained from the hospital database and patient files. Intraoperative complications were extracted from the operation reports. Postoperative imaging, particularly CT-angiography, was conducted on all patients between the first and fifth day after surgery to assess the outcome of the aneurysm treatment. Postoperative complications were extracted from clinical records. Long-term outcomes, including 30-day mortality rates, mRS scores at 1 and 12 months, aneurysm recurrence, and the necessity for additional treatment, were assessed during routine follow-up appointments at our specialized outpatient clinic.

### 2.5. Statistical Analysis

Differences between the pterional and supraorbital cohorts were analyzed using Student’s *t*-test for independent samples or the Wilcoxon rank-sum test for paired samples. Categorical variables were evaluated with the exact Chi-squared test. Descriptive statistics were provided for patient characteristics, aneurysm-related factors, treatment-related factors, complications, and outcomes. Nominal variables were displayed as both absolute and relative frequencies, while continuous variables were conveyed as means with standard deviation.

In all instances, we calculated exact *p*-values; we regarded *p* ≤ 0.05 as statistically significant.

Analyses were conducted using the statistical software IBM SPSS Statistics Version 28 (Release 28.0.0.0, 2022. Armonk, NY, USA: International Business Machines Corporation).

## 3. Results

### 3.1. Demographic Characteristics

The study included 170 patients in the pterional group and 71 patients in the supraorbital group. The median age at surgery was 56 years, with a higher proportion of females in both groups. Upon admission, 159 of 170 patients (93.5%) in the pterional group had a modified Rankin Scale (mRS) score between 0 and 2, while 11 patients (6.5%) scored between 3 and 4 (5.9% with mRS 3, 0.6% with mRS 4). In the supraorbital group, 68 of 71 patients (95.8%) presented with mRS scores of 0 to 2, and 3 patients (4.2%) had a score of 3.

The American Society of Anesthesiologists Physical Status Classification (ASA classification) was recorded for 140 out of 170 patients (82.4%) in the pterional group and for 66 out of 71 patients (93.0%) in the supraorbital group. In the pterional group, 18 patients were classified as ASA 1 (10.6%), 80 as ASA 2 (47.1%), 39 as ASA 3 (22.9%), and 2 as ASA 4 (1.2%). In the supraorbital group, 11 patients were ASA 1 (15.5%), 40 ASA 2 (56.3%), 14 ASA 3 (19.7%), and 2 ASA 4 (2.8%).

The baseline demographic and clinical characteristics showed no substantial differences between the two groups (Table 1).

### 3.2. Aneurysm and Approach Related Characteristics

CSF was released either from the basal cisterns/proximal Sylvian fissure or from the distal Sylvian fissure after dural opening. In the pterional approach, drainage was mainly from the basal cisterns/proximal Sylvian fissure (113 cases, 66.5%), while in 57 cases (33.5%) CSF release was achieved primarily via opening of the distal Sylvian fissure. In the supraorbital approach, CSF was almost always released from the basal cisterns/proximal Sylvian fissure (64 cases, 90.1%) and only rarely via the distal Sylvian fissure (7 cases, 9.9%). The association between surgical approach and CSF drainage site was significant (*p* < 0.001).

The distribution of treated aneurysms demonstrated notable disparities between the two techniques. The pterional technique exhibited a considerably higher occurrence of aneurysms at the middle cerebral artery (MCA), with 145 aneurysms (79.2%) in contrast to 35 aneurysms (42.2%) in the supraorbital technique. Conversely, the supraorbital technique had a greater percentage of treated aneurysms at the internal carotid artery (ICA), with 15 aneurysms (18%) versus 9 aneurysms (4.9%) in the pterional technique. Furthermore, anterior communicating artery (ACOM) aneurysms were more prevalent in the supraorbital technique, with 29 aneurysms (34.9%) compared to 23 aneurysms (12.5%) in the pterional group. These differences were statistically significant (*p* < 0.001). Table 2 outlines the anatomical distribution of the treated aneurysms.

Morphological aneurysm parameters (width, height, aspect ratio) and surgical approach related characteristics are summarized in Table 3. Significant differences were observed for aneurysm width (*p* < 0.01), height (*p* = 0.02), aspect ratio (*p* = 0.013), and craniotomy area (*p* < 0.01). Differences in operative duration (*p* = 0.151) and temporary clipping time (*p* = 0.836) were not statistically significant.

The surgical strategies used in both groups, including clipping, wrapping, and combined techniques, are outlined in Table 4. While combined strategies were more frequent in the supraorbital group, the differences between the groups did not reach statistical significance (*p* = 0.506).

The average length of hospital stay for the pterional approach was 8.76 days (minimum 5, maximum 28, SD 3.93), while the supraorbital approach had an average of 7.96 days (minimum 5, maximum 44, SD 5.37). This difference was statistically significant (*p* = 0.001).

Concerning complete aneurysm occlusion as assessed by postoperative CTA, 143 patients (84.1%) in the pterional cohort achieved total occlusion, and 19 (11.2%) had partial occlusion; data were missing for 4.7%. In the supraorbital cohort, 62 patients (87.3%) showed complete occlusion, 3 (4.2%) had partial occlusion, and 8.5% lacked data. This difference was not statistically significant (*p* = 0.077).

Incomplete occlusion occurred predominantly in aneurysms treated with wrapping or combined techniques, whereas clipping alone resulted in complete occlusion in 94.7% of cases; this association was statistically significant (*p* < 0.001).

Aneurysm recurrence during follow-up was observed in 6 cases (3.5%) in the pterional group and none in the supraorbital group (*p* = 0.122). Retreatment was required in 2 cases (1.2%) of the pterional group, and in none in the supraorbital group (*p* = 0.499).

The patient in the pterional group underwent flow-diverter implantation with adjunctive coiling for a recurrent 9 mm MCA bifurcation aneurysm, with complete occlusion confirmed on follow-up CTA. The patient in the supraorbital group required re-craniotomy and re-clipping for a progressive broad-based ACOM remnant, and subsequent CTA demonstrated complete occlusion.

### 3.3. Complications

In terms of significant intraoperative complications (surgery-related issues or intraoperative aneurysm rupture), 15 patients (8.8%) in the pterional cohort faced serious complications. In contrast, the supraorbital cohort showed 19 instances (26.8%) of severe intraoperative complications. The disparity between the two cohorts was statistically significant (*p* < 0.001).

Among these severe intraoperative complications, intraoperative aneurysm rupture occurred notably more often in the supraorbital cohort than in the pterional cohort (*p* < 0.001). Specifically, 11 out of 170 patients (6.5%) in the pterional cohort and 19 out of 71 patients (26.8%) in the supraorbital cohort had an intraoperative aneurysm rupture.

Further characterizing these events, intraoperative ruptures were classified as either premature or occurring after proximal control. Premature rupture was observed in 9 cases overall, including 4 of 170 patients in the pterional cohort (2.35%) and 5 of 71 patients in the supraorbital cohort (7.0%). In contrast, 21 ruptures occurred after proximal control, comprising 8 of 170 pterional cases (4.71%) and 13 of 71 supraorbital cases (18.3%). The distribution of rupture timing did not differ significantly between approaches (*p* = 0.745).

Concerning perioperatively reported complications, the pterional approach recorded 53 complications in 39 patients (22.9%). Conversely, the supraorbital approach recorded 31 incidents in 25 patients (35.2%). The difference observed between the two cohorts was statistically significant (*p* = 0.049).

Postoperative hyp-/anosmia was significantly more prevalent in the supraorbital cohort (*p* = 0.013), affecting 6 out of 71 patients (8.5%), compared to 3 out of 170 patients (1.8%) in the pterional cohort.

Ninety complications in 53 patients were reported during follow up for the pterional technique, with 42 patients (24.7%) being unaccounted for. Twenty-five complications in 20 patients were reported during follow up for the supraorbital technique, with 12 (16.9%) patients lost to follow-up. The disparity between the two groups did not reach statistical significance (*p* = 0.331).

A noticeable atrophy of the M. temporalis was more prevalent in the pterional group (*p* = 0.029), impacting 14 out of 128 patients (10.9%), whereas only 1 out of 59 patients (1.7%) in the supraorbital group experienced this issue. The others were lost to follow up (pterional: 42, supraorbital: 12).

Furthermore, chewing difficulties and/or transient jaw lock occurred more frequently in the pterional group (*p* = 0.002), affecting 23 out of 128 patients (18%), in contrast to 1 out of 59 patients (1.7%) in the supraorbital group. Again, the remaining patients were lost to follow-up (pterional: 42, supraorbital: 12).

**Figure 2:**
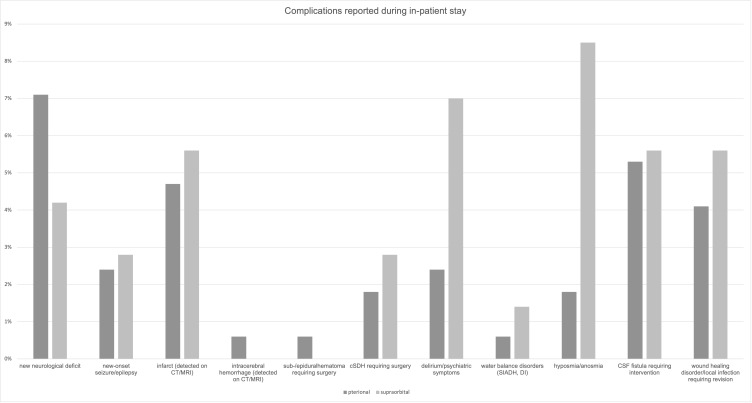
Complications reported during in-patient stay. Abbreviations: CT, computed tomography; MRI, magnetic resonance imaging; cSDH, chronic subdural hematoma; SIADH, syndrome of inappropriate antidiuretic hormone secretion; DI, diabetes insipidus; CSF, cerebrospinal fluid.

**Figure 3:**
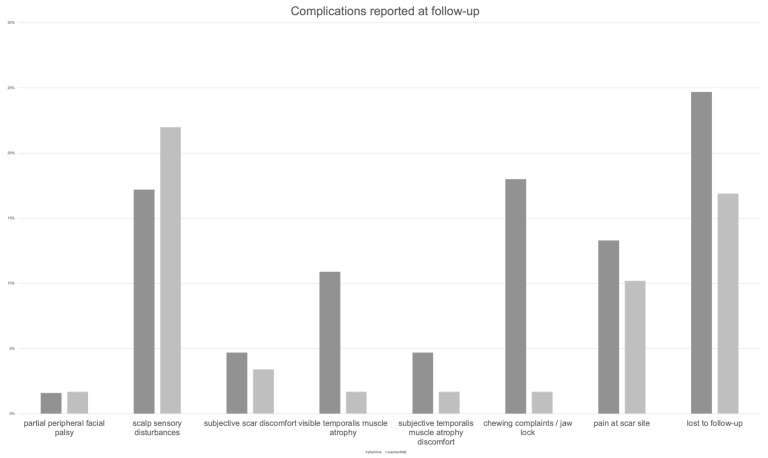
Complications reported at follow-up.

A comprehensive account of complications for both approaches is detailed in Table 5 and Figure 2 and Figure 3.

Regarding 30-day-mortaility, the pterional approach had 1 case (0.6%) of mortality from 170 cases, while the supraorbital approach also reported 1 case (1.4%) from 71 cases. The difference between the two groups was not statistically significant (*p* = 0.521).

The cause of death in the supraorbital case was an anterior and medial cerebral infarction. In the pterional case, mortality resulted from multiple cerebral infarctions.

### 3.4. Outcome

The functional outcomes of individuals who underwent surgical interventions via pterional and supraorbital approaches were evaluated utilizing the modified Rankin Scale (mRS) at intervals of one month and twelve months subsequent to the surgical procedures.

#### 3.4.1. One Month Postoperatively

In the pterional cohort (*n* = 170), mRS data was obtainable for 157 participants, while 13 individuals (7.6%) could not be contacted for follow-up. Among the cases with available information, 141 individuals (89.8%) attained a favorable outcome (mRS 0–2), whereas 15 individuals (9.6%) encountered moderate-severe disability (mRS 3–4).

In the supraorbital group (*n* = 71), mRS information was accessible for 69 patients, with 2 patients (2.9%) lost to follow-up. Among the cases with available data, 60 patients (87%) achieved a favorable outcome (mRS 0–2), while 8 patients (11.6%) faced moderate-severe disability (mRS 3–5). Statistical analysis indicated no significant difference between the two groups (*p* = 0.707).

#### 3.4.2. Twelve Months Postoperatively

In the pterional cohort (*n* = 170), mRS data were accessible for 107 patients, whereas 63 patients (37.1%) were not followed up with. Among the patients with available data, 94 individuals (87.9%) experienced a favorable outcome (mRS 0–2), while 13 patients (12.1%) exhibited moderate-severe disability (mRS 3–4).

In the supraorbital cohort (*n* = 71), mRS data were accessible for 53 patients, while 18 patients (25.4%) were not followed up with. Among the patients with available data, 49 individuals (92.5%) demonstrated a favorable outcome (mRS 0–2), while 4 patients (7.5%) showed moderate disability (mRS 3).

No significant difference was observed between the two cohorts at 12 months (*p* = 0.899).

**Figure 4:**
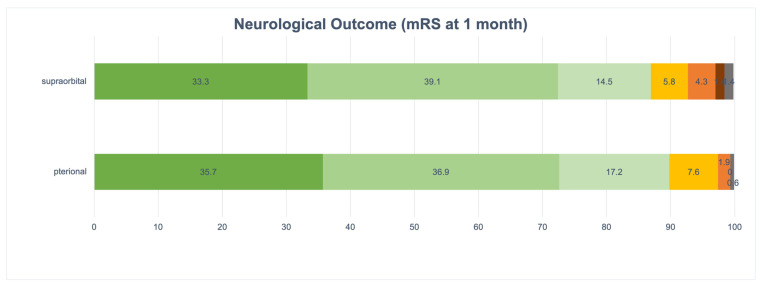
Neurological Outcome (mRS at 1 month). Abbreviations: mRS, modified Rankin Scale.

**Figure 5:**
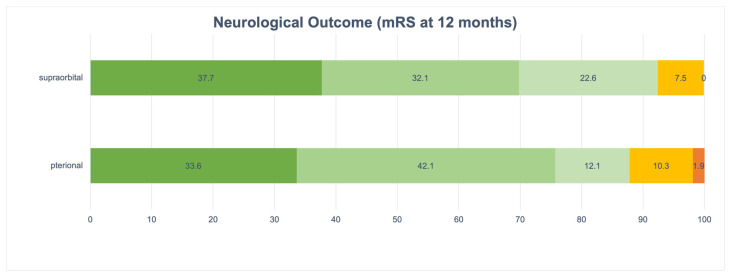
Neurological Outcome (mRS at 12 months). Abbreviations: mRS, modified Rankin Scale.

The comprehensive distribution of mRS scores at both time points is detailed in Table 6 and Figure 4 and Figure 5.

## 4. Discussion

In this study, we analyzed the pterional and supraorbital techniques for addressing unruptured intracranial aneurysms, focusing on complication rates, mortality, and functional results. Up to now, the majority of studies, systematic reviews, and meta-analyses have assessed these two surgical methods in diverse groups that include both ruptured and unruptured aneurysms [8,12,15,16,17,18,19,20,21]. Nevertheless, to our knowledge, our study is only the second after Shin et al. [22] to specifically explore these two techniques in the context of unruptured aneurysms. With a total of 241 patients, our study constitutes the largest cohort investigation to date centered exclusively on incidental, unruptured aneurysms, facilitating a more focused evaluation of surgical risks and benefits for this particular patient group.

Our study demonstrates that aneurysm characteristics such as location, segmental distribution, and spatial orientation significantly influence the choice of surgical approach. These anatomical factors suggest that preoperative aneurysm features played a crucial role in determining the optimal surgical route. This aligns with earlier studies emphasizing the importance of aneurysm location in surgical planning [12,15,19]. Furthermore, differences in aneurysm size and shape parameters—including width, height, and aspect ratio—between the groups highlight how anatomical complexity affects the selection of the surgical technique.

One of the most important discoveries in our research was the notably elevated occurrence of severe intraoperative complications within the supraorbital group, mainly due to a higher rate of intraoperative aneurysm ruptures. This finding aligns with the observations made by Madhugiri et al. [19], who similarly noted an increased rate of intraoperative rupture in ruptured aneurysms managed through the supraorbital method. The limited surgical corridor and constrained maneuverability associated with the supraorbital technique may heighten this risk, especially in situations where aneurysm exposure is inadequate. Conversely, Brown et al. [8] found no significant disparities between the two methods in terms of clipping rates, intraoperative rupture incidents, or postoperative complications, indicating a comparable overall safety profile. Nevertheless, our findings suggest that although the supraorbital approach provides minimally invasive access, judicious patient selection is essential to reduce intraoperative risks.

On the other hand, serious postoperative complications displayed only marginal statistical relevance (*p* = 0.049), indicating a trend instead of a clear distinction. This observation aligns with earlier research that revealed no significant discrepancies in postoperative complication rates between the two methods [8,20]. Nevertheless, certain complications were found to differ notably. In our investigation, the supraorbital approach was linked to a considerably elevated incidence of hyp-/anosmia, probably due to the surgical path passing through the frontal base. This is a recognized risk tied to the supraorbital technique, as the olfactory nerve may be impacted by the manipulation of structures in the frontal region.

In contrast, access-associated complications were more common in the pterional group, exhibiting notably higher instances of temporalis muscle atrophy, difficulties in chewing, and temporary jaw lock. These observations are corroborated by previous research highlighting the impacts of extensive soft tissue dissection during pterional craniotomies [8,19].

Our findings also indicated that the supraorbital technique was linked to a notably shorter duration of in-patient stay, which is consistent with reports from Madhugiri et al. [19] and Shin et al. [22]. Shin et al. [22] particularly focused on unruptured aneurysms of the supraclinoid ICA and noted not just a decrease in operative time but also a diminished requirement for intraoperative blood transfusions and a reduced occurrence of postoperative epidural hematomas in the supraorbital cohort. This implies that the supraorbital method might provide perioperative benefits in certain cases. Likewise, Pichugin et al. [15] noted less blood loss, a lower rate of postoperative neurological deficits, and a decreased frequency of ischemic and hemorrhagic complications in the supraorbital cohort. Nevertheless, these benefits need to be considered alongside the heightened risk of intraoperative rupture noted in our investigation, as well as in the study conducted by Madhugiri et al. [19].

Despite these variations, the functional results were similar across the two methods. At both 1 month and 12 months after surgery, modified Rankin Scale (mRS) scores indicated no substantial differences, aligning with findings from Brown et al. [8] and Lan et al. [20]. Wu et al. [21] noted that the supraorbital technique yielded improved outcomes for unruptured middle cerebral artery aneurysms, while no significant differences were detected between the methods for ruptured aneurysms. In our research, both cohorts attained comparable rates of positive outcomes at 1 month (mRS 0–2: 89.8% for pterional versus 87% for supraorbital, *p* = 0.707) and 12 months (87.9% for pterional versus 92.5% for supraorbital, *p* = 0.899). Nevertheless, the greater rate of loss to follow-up in the pterional group at 12 months (37.1% versus 25.4%) restricts definitive long-term conclusions.

### Clinical Implications

Our research emphasizes the importance of a customized strategy when choosing the most suitable surgical method for unruptured intracranial aneurysms. Although the supraorbital technique provides benefits such as minimally invasive access, shorter hospital stays, and fewer access-related complications, it also presents a greater chance of intraoperative rupture and olfactory issues. On the other hand, the pterional approach provides a wider surgical exposure, potentially reducing the risk of intraoperative rupture, but is associated with a higher incidence of access-related complications such as temporalis muscle atrophy and chewing difficulties.

Taking these factors into account, patient-specific elements—like aneurysm location, shape, and unique anatomical differences—must be thoroughly evaluated. In situations where limited exposure suffices and the intraoperative rupture risk is considered low, the supraorbital technique might be advantageous. Conversely, for cases necessitating a larger surgical pathway, especially in the case of intricate aneurysms, the pterional approach continues to be the preferred choice.

## 5. Study Limitations

This study is limited by its retrospective design, which inherently introduces selection bias as the choice of surgical approach was influenced by aneurysm characteristics. Loss to follow-up, particularly in the pterional group, also weakens the long-term outcome analysis. Although this is the largest cohort comparing these techniques for unruptured aneurysms, larger multicenter studies with standardized criteria are needed to confirm the findings. Future research should include prospective trials to improve patient selection and evaluate intraoperative imaging and navigation to reduce complications.

## 6. Conclusions

This study is the second after Shin et al. comparing pterional and supraorbital approaches for unruptured intracranial aneurysms, involving the largest cohort to date. Both approaches achieve good functional outcomes but differ in risks: the supraorbital is less invasive with faster recovery but higher risk of rupture and olfactory dysfunction; the pterional provides better exposure but more temporalis muscle atrophy and chewing difficulties.

## Figures and Tables

**Table 1:** Demographic characteristics.

Demographic Characteristics	Pterional *n* (%)	Supraorbital *n* (%)	*p*-Value
Number of patients	170 (70.5%)	71 (29.5%)	
Age at admission (median)	56	56	0.983
Sex			0.265
Male	42 (24.7%)	23 (32.4%)	
Female	128 (75.3%)	48 (67.6%)	
mRS at admission			0.690
No symptoms (0)	67 (39.4%)	30 (42.3%)	
No significant disability (1)	71 (41.8%)	28 (39.4%)	
Slight disability (2)	21 (12.4%)	10 (14.1%)	
Moderate disability (3)	10 (5.9%)	3 (4.2%)	
Moderate severe disability (4)	1 (0.6%)	0 (0%)	
ASA classification			0.428
ASA 1	18 (10.6%)	11 (15.5%)	
ASA 2	80 (47.1%)	40 (56.3%)	
ASA 3	39 (22.9%)	14 (19.7%)	
ASA 4	2 (1.2%)	2 (2.8%)	
Not recorded	30 (17.6%)	5 (7%)	

Abbreviations: *n*, number of patients; mRS, modified Rankin Scale; ASA, American Society of Anesthesiologists Physical Status Classification.

**Table 2:** Distribution of treated aneurysms according to vascular segment and approach.

Distribution of Treated Aneurysms	Pterional *n* (%)	Supraorbital *n* (%)	*p*-Value
			<0.001
Number of aneurysms	183	83	
ACA	28 (15.3%)	30 (36.1%)	
A1	5 (2.7%)	1 (1.2%)	
ACOM	23 (12.5%)	29 (34.9%)	
ICA	9 (4.9%)	15 (18%)	
C5	0 (0%)	1 (1.2%)	
C6	1 (0.5%)	7 (8.4%)	
C7	6 (3.3%)	4 (4.8%)	
C7/M1	0 (0%)	1 (1.2%)	
C7/PCOM	2 (1%)	2 (2.4%)	
MCA	145 (79.2%)	35 (42.2%)	
M1	61 (33.3%)	14 (16.9%)	
M1/M2	69 (37.7%)	17 (20.5%)	
M2	14 (7.7%)	4 (4.8%)	
M2/M3	1 (0.5%)	0 (0%)	
PCOM	1 (0.5%)	3 (3.6%)	

Abbreviations: *n*, number of aneurysms; ACA, anterior cerebral artery; A1, A1-segment of anterior cerebral artery; ACOM, anterior communicating artery; ICA, internal carotid artery (Bouthillier classification); C5, C5-segment of internal carotid artery; C6, C6-segment of internal carotid artery; C7, C7-segment of internal carotid artery; C7/M1, transition segment between C7-segment and M1-segment of middle cerebral artery; C7/PCOM, transition segment between C7-segment and posterior communicating artery; MCA, middle cerebral artery; M1, M1-segment of middle cerebral artery; M1/M2, transition segment between M1 and M2; M2, M2-segment of middle cerebral artery; M2/M3, transition segment between M2 and M3; PCOM, posterior communicating artery.

**Table 3:** Morphological aneurysm parameters and surgical approach-related characteristics.

Aneurysm and Approach-Related Characteristics	Pterional Mean ± SD	Supraorbital Mean ± SD	*p*-Value
Aneurysm characteristics			
Aneurysm width (mm)	6.88 ± 3.23	5.04 ± 2.20	<0.01
Aneurysm height (mm)	7.17 ± 3.41	5.50 ± 2.13	0.02
Aspect Ratio	1.65 ± 0.64	1.37 ± 0.49	0.013
Approach-related characteristics			
Craniotomy area (cm^2^)	20.35 ± 9.17	4.98 ± 1.36	<0.01
Operative time (min)	148.03 ± 42.19	141.62 ± 25.73	0.151
Temporary clipping (min)	2.92 ± 4.23	3.10 ± 4.99	0.836

Abbreviations: mm, millimeter; cm^2^, square centimeter; min, minute.

**Table 4:** Surgical strategies.

Surgical Strategies	Pterional *n* (%)	Supraorbital *n* (%)	*p*-Value
Number of aneurysms	183	83	0.506
Clipping only	143 (78.1%)	59 (71.1%)	
Wrapping only	6 (3.3%)	2 (2.4%)	
Clipping and Wrapping	33 (18%)	21 (25.3%)	
Attempt/probatory	1 (0.5%)	1 (1.2%)	

**Table 5:** Complications reported during in-patient stay and complications reported during follow-up.

Complications	Pterional *n* (%)	Supraorbital *n* (%)	*p*-Value
Complications reported during in-patient stay	53	31	0.049
New neurological deficit	12 (7.1%)	3 (4.2%)	0.563
New-onset seizure/epilepsy	4 (2.4%)	2 (2.8%)	1.000
Infarct (detected on CT/MRI)	8 (4.7%)	4 (5.6%)	0.752
Intracerebral hemorrhage (detected on CT/MRI)	1 (0.6%)	0 (0%)	1.000
Sub-/epidural hematoma requiring surgery	1 (0.6%)	0 (0%)	1.000
CSDH requiring surgery	3 (1.8%)	2 (2.8%)	0.633
Delirium/psychiatric symptoms	4 (2.4%)	5 (7%)	0.129
Water balance disorders (SIADH, DI)	1 (0.6%)	1 (1.4%)	0.500
Hyposmia/anosmia	3 (1.8%)	6 (8.5%)	0.021
CSF fistula requiring intervention	9 (5.3%)	4 (5.6%)	1.000
Wound healing disorder/local Infection requiring revision	7 (4.1%)	4 (5.6%)	0.736
Complications reported during follow-up	90	25	0.331
Partial peripheral facial palsy	2 (1.6%)	1 (1.7%)	1.000
Scalp sensory disturbances	22 (17.2%)	13 (22%)	0.543
Subjective scar discomfort	6 (4.7%)	2 (3.4%)	1.000
Visible temporalis muscle atrophy	14 (10.9%)	1 (1.7%)	0.029
Subjective temporalis muscle atrophy discomfort	6 (4.7%)	1 (1.7%)	0.432
Chewing complaints/jaw lock	23 (18%)	1 (1.7%)	0.002
Pain at scar site	17 (13.3%)	6 (10.2%)	0.635
Lost to follow-up	42 (24.7%)	12 (16.9%)	

Abbreviations: CT, computed tomography; MRI, magnetic resonance imaging; SIADH, syndrome of inappropriate antidiuretic hormone secretion; DI, diabetes insipidus; CSF, cerebrospinal fluid.

**Table 6:** Neurological Outcome (mRS) at 1 and 12 months postoperatively.

Neurological Outcome	Pterional *n* (%)	Supraorbital *n* (%)	*p*-Value
Neurological Outcome (mRS at 1 month)			0.707
No symptoms (0)	56 (35.7%)	23 (33.3%)	
No significant disability (1)	58 (36.9%)	27 (39.1%)	
Slight disability (2)	27 (17.2%)	10 (14.5%)	
Moderate disability (3)	12 (7.6%)	4 (5.8%)	
Moderately severe disability (4)	3 (1.9%)	3 (4.3%)	
Severe disability (5)	0 (0%)	1 (1.4%)	
Dead (6)	1 (0.6%)	1 (1.4%)	
Not recorded	13 (7.6%)	2 (2.9%)	
Neurological Outcome (mRS at 12 months)			0.899
No symptoms (0)	36 (33.6%)	20 (37.7%)	
No significant disability (1)	45 (42.1%)	17 (32.1%)	
Slight disability (2)	13 (12.1%)	12 (22.6%)	
Moderate disability (3)	11 (10.3%)	4 (7.5%)	
Moderately severe disability (4)	2 (1.9%)	0 (0%)	
Severe disability (5)	0 (0%)	0 (0%)	
Dead (6)	0 (0%)	0 (0%)	
Not recorded	63 (37.1%)	18 (25.4%)	

Abbreviations: mRS, modified Rankin Scale.

## Data Availability

The data presented in this study are not publicly available due to institutional data protection policies but are available from the corresponding author upon reasonable request.
